# Uncovering the Correlation between COVID-19 and Neurodegenerative Processes: Toward a New Approach Based on EEG Entropic Analysis

**DOI:** 10.3390/bioengineering10040435

**Published:** 2023-03-29

**Authors:** Andrea Cataldo, Sabatina Criscuolo, Egidio De Benedetto, Antonio Masciullo, Marisa Pesola, Raissa Schiavoni

**Affiliations:** 1 Department of Engineering for Innovation, University of Salento, 73100 Lecce, Italy; 2 Department of Electrical Engineering and Information Technology (DIETI), University of Naples Federico II, 80125 Naples, Italy

**Keywords:** COVID-19, Alzheimer’s disease, biomedical, mild cognitive impairment, electroencephalography, EEG, entropy, brain, Multiscale Fuzzy Entropy, neurodegenerative, olfactory dysfunction

## Abstract

COVID-19 is an ongoing global pandemic caused by the severe acute respiratory syndrome coronavirus 2 (SARS-CoV-2) virus. Although it primarily attacks the respiratory tract, inflammation can also affect the central nervous system (CNS), leading to chemo-sensory deficits such as anosmia and serious cognitive problems. Recent studies have shown a connection between COVID-19 and neurodegenerative diseases, particularly Alzheimer’s disease (AD). In fact, AD appears to exhibit neurological mechanisms of protein interactions similar to those that occur during COVID-19. Starting from these considerations, this perspective paper outlines a new approach based on the analysis of the complexity of brain signals to identify and quantify common features between COVID-19 and neurodegenerative disorders. Considering the relation between olfactory deficits, AD, and COVID-19, we present an experimental design involving olfactory tasks using multiscale fuzzy entropy (MFE) for electroencephalographic (EEG) signal analysis. Additionally, we present the open challenges and future perspectives. More specifically, the challenges are related to the lack of clinical standards regarding EEG signal entropy and public data that can be exploited in the experimental phase. Furthermore, the integration of EEG analysis with machine learning still requires further investigation.

## 1. Introduction and Motivation of the Work

The coronavirus disease 2019 (COVID-19) is an infectious disease caused by the severe acute respiratory syndrome coronavirus 2 (SARS-CoV-2). This virus can cause a wide spectrum of symptoms [1]. Although it primarily attacks the respiratory tract, the inflammation can act heterogeneously on almost any organ, leading to kidney, gastrointestinal, pulmonary, cardiovascular, and even serious cognitive problems [2]. In fact, it can attack the central nervous system (CNS) causing chemo-sensory deficits such as anosmia, encephalitis, cerebrovascular disorders, or brain fog [3,4]. An increasing number of studies [5,6] indicate that the SARS-CoV-2 virus has the capability to overcome the blood–brain barrier (BBB) and compromise neurons, potentially causing permanent damage. The impairment of the BBB allows the entry of toxic substances, inflammatory agents, and immune cells into the brain, ultimately causing neuronal deterioration and cognitive decline. The disruption of the BBB is thought to play a key role in the development and progression of many neurological conditions. Hence, the existence of a common mechanism involving the BBB and neuro-inflammation implies a potential connection between COVID-19 and neurodegenerative diseases such as dementia, Parkinson’s, and Alzheimer’s disease (AD) [7,8].

In more detail, the SARS-CoV-2 virus directly invades the CNS due to the interaction between the SARS-CoV-2 spike protein and angiotensin-converting enzyme 2 (ACE-2), which is a membrane carboxypeptidase and is considered the main site of entry of SARS-CoV-2 into cells [9]. As a consequence, greater soluble ACE-2 levels in the brain tissues may enhance the risk of SARS-CoV-2 infection in CNS tissues. ACE-2 is mostly expressed on neurons in the temporal lobe and hippocampus, areas which are involved in the pathophysiology of AD [10], the most common age-related neurological disorder affecting about 55 million people globally. In this regard, studies were conducted to examine whether ACE-2 levels were post-mortem-altered in the parietal cortex areas of AD subjects. It was found that the ACE-2 level was higher [11] in those with a neuropathological diagnosis of AD compared to age-matched controls. This aspect could be a risk factor for COVID-19 interacting with the virus spike protein [12]. SARS-CoV-2 could accelerate the development of neurodegenerative disorders and potentially induce a worsening cognitive decline in patients with AD [13,14,15,16,17,18], which, in the early stage, manifests as a mild cognitive impairment (MCI), which includes slight memory impairment as the most typical symptom. In a recent study [19], researchers examined the levels of several serum biomarkers (typically associated with neurodegenerative disorders) in COVID-19 patients without prior history of dementia versus non-COVID-19 healthy, MCI, and AD subjects. The levels of the considered serum biomarkers in COVID-19 patients were comparable with MCI and AD patients. In addition, recent studies have shown that the apolipoprotein E ε4 allele (APOE4) is directly linked to similar genetic factors implicated in COVID-19 and AD [20,21,22]. These investigations suggest that neurological mechanisms of protein interactions during COVID-19 are similar to those exhibited in AD conditions.

On the other hand, recently, long-term neurological effects due to SARS-CoV2 infection have been recognized [23] and associated with inflammatory processes, which are central to AD. This suggests a bidirectional relationship between the two pathologies. Several studies support the theory of neurotropic involvement of SARS-CoV-2 [24]. As a result, much more attention should be paid to the risk of neurological involvement in patients with COVID-19. It is well known that a neurotropic virus can result in the alteration of the EEG signal [25], such as in the case of the Japanese encephalitis virus, which causes an alteration in the EEG delta band and nonresponsive activity associated with the alpha band [26]. Another example is the poliovirus, which causes slow-wave EEG activity [27]. In addition, another neurotropic virus, such as the Measles morbillivirus, may result in lesions of the subcortical white matter and the occurrence of periodic high-amplitude slow EEG waves in case of non-vaccination [28]. Furthermore, echovirus is associated with a characteristic EEG pattern and is characterized by convulsions [29]. Similarly, the potential neurotropic nature of SARS-CoV-2 could be associated with some abnormalities in the EEG patterns. For this reason, it is important to identify and quantify the common features between AD and COVID-19 and the possible similarity of the effects that they imply on the human nervous system. Should these commonalities be consistent, in fact, then neurodegenerative processes would be added to the list of severe, long-term effects caused by the SARS-CoV-2 virus.

Although several aforementioned studies are underway to prove such an impactful statement at a physiological level, the present evidence only partially corroborates the association between COVID-19 and the manifestations of neurodegenerative diseases. The absence of quantitative biomarkers for tracking disease progression represents an issue in understanding potentially shared mechanisms between these pathologies [17]. In addition to that, reliable biomarkers could help clinicians effectively manage the impact of an acute SARS-CoV-2 infection on brain dynamics, particularly in situations where the risk of triggering or worsening neurological conditions is elevated [16]. COVID-19 has been shown to cause neurological symptoms [3,4], such as confusion, delirium, and stroke, particularly in older adults and those with pre-existing conditions. Such symptoms could potentially contribute to the development or progression of Alzheimer’s disease in some individuals. However, it is crucial to note that this remains an active research area, and further studies are necessary to comprehend the potential long-term consequences of COVID-19 on cognitive health.

One intriguing aspect identified in the literature is that both SARS-CoV-2 infections and neurodegenerative diseases [30,31] (AD in particular) may lead to an impairment of the olfactory system. Anosmia, the loss of sense of smell, is one of the first and most frequent symptoms of COVID-19, and an estimated 85% to 98% of patients experience some degree of olfactory dysfunction [32]. Although this symptom typically resolves spontaneously within two weeks in the majority of cases, a substantial number of patients may suffer from persistent post-COVID-19 olfactory impairment. While potentially affecting millions of people, this symptom is also associated with a range of debilitating psycho-social effects, including depression and cognitive impairment. Since this phenomenon may be indicative of complex cerebral mechanisms involved in cognitive decline, such as localized nasal dysfunctions, investigating potential biomarkers for changes in brain dynamics could be a worthwhile pursuit [17]. Hence, the analysis of electroencephalographic (EEG) signals (when they are subjected to olfactory tasks) could be a promising strategy to investigate if (and to what extent) SARS-CoV-2 infection and AD are related. The EEG signal analysis could help in the exploration of common features among patients with COVID-19 and AD, with the advantages of non-invasiveness and cost-effectiveness, compared to other neuroimaging techniques. Non-linear analyses (particularly among advanced EEG signal-processing techniques) investigate the complex emergent phenomena underlying chaotic dynamical systems, providing the possibility to analyze the strong complexity and irregularity of neuronal activities of the brain. These analyses are of extreme importance in order to identify the process of cognitive impairment [33]. Indeed, the complexity measures of EEG signals could enhance and provide more reliable results than traditional EEG analysis techniques (e.g., event-related potential, time, and frequency analysis) in the studies of psycho-pathological conditions [34] and the diagnosis of disorders [35]. Linearity measures, such as power spectral analyses, assume the stationary of the system. However, neuronal processes in the brain are known to be non-stationary and exhibit complex and time-evolving behavior [36,37] and, thus, non-linear dynamical analyses may be more suitable for studying brain dynamics and understanding neural processes, particularly in neurological conditions, such as Alzheimer’s disease, which weaken inter-neuronal connections. This is because non-linear analyses assume statistical non-stationary as an inherent characteristic of neural signals and can provide more reliable insights into the changes in the complexity of EEG signals resulting from these disorders [35,38].

Recently, EEG complexity measures have been employed in studies focused on COVID-19. Pastor et al. [39] calculated Shannon’s spectral entropy (SSE) and synchronization by Pearson’s correlation coefficient by comparing patients with COVID-19 and those with infectious toxic or post-cardiorespiratory arrest encephalopathy. The results suggested that SSE was higher and hemispheric connectivity was lower for COVID-19 patients. However, the literature lacks studies that relate to COVID-19 and brain complexity. In this regard, entropy-based analysis in COVID-19 patients could confirm the connection with AD.

Based on these considerations, this work outlines the implementation of an entropy-based approach for the detection of possible common EEG features between the early stages of AD, such as MCI conditions, and patients suffering from long-COVID, a persistent condition characterized by symptoms such as persistent effort, weakness, muscle and joint pain, headaches, confusion, and smell disorders, even weeks or months after acquiring SARS-CoV-2 infection. In particular, at the current state of the art, the possible neurodegenerative action of COVID-19 is the main subject of intensive studies and research. In this perspective, At the current state of the art, intensive studies and research are focused on investigating the possible neurodegenerative effects of COVID-19. It is of great interest to explore its mechanisms at the brain level by comparing them with established neurological diseases such as Alzheimer’s. Such a perspective could lead to a better understanding of the neurodegenerative phenomena associated with COVID-19, enable a comparison of its severity with those related to AD, and identify possible treatments. In particular, the multi-scale fuzzy entropy (MFE) technique has great potential for this purpose, as explained in the following sections. Because both AD and COVID-19 exhibit olfactory deficits, this work employs an olfactory recognition task during the experimental process to investigate common EEG features between COVID-19 and AD in response to an olfactory stimulus.

This work is organized as follows. Section 2 provides an overview of the state-of-the-art solutions for neurodegenerative disease diagnostics based on the study of brain complexity. Section 3 presents the basic theoretical background behind the proposed method and possible algorithm implementation. Section 4 analyzes the future perspectives and open challenges for entropy applications on EEG signals (for the early detection of neurological deficits). Finally, the conclusions are outlined in Section 5.

## 2. Review of Complexity Measures for the Analysis of EEG Signals

Brain activity is a complex phenomenon that involves non-linear and dynamic processes and is characterized by behavior that is neither completely regular nor completely random [40]. The organization and interactions of neurons in the brain are highly intricate, resulting in the generation of a coherent and multimodal scene through the integration of brain signals from functionally specialized groups of neurons located in various brain areas. Additionally, the combination of continuous spontaneous brain activity with evoked signals from the environment adds to the complexity of the neural system.

The concept of neural complexity entails the coexistence of functional specialization and integration [40], which are two crucial aspects of the organization and functioning of the human brain. Functional specialization refers to the idea that different regions of the brain specialize in processing specific types of information or performing specific functions. This specialization arises from the fact that different brain regions have distinct anatomical and physiological properties that make them better suited for certain types of processing. On the other hand, functional integration refers to the coordination and communication between different specialized regions of the brain to achieve more complex functions. This integration is essential because most cognitive tasks require the involvement of multiple brain regions working together in a coordinated manner. For example, language processing involves the activation of several different brain areas, including those involved in sound processing, grammar, and meaning. Overall, the concept of neural complexity acknowledges that the brain is not just a collection of isolated regions, but rather a highly interconnected and integrated network of specialized areas that work together to support complex cognitive functions.

However, these underlying physiological processes are not always discernible through neuroimaging techniques. In electroencephalography (EEG), the voltage signal reflects the activity of populations of neurons. The field of cognitive electrophysiology is concerned with investigating the relationship between cognitive functions, such as perception, memory, language, emotions, and behavioral control, and the electrical activity of neural populations [41]. Complexity measures, which assess the degree of interaction among functional brain networks across local and global scales, can provide a deeper understanding of brain function and potential alterations in various neurological conditions.

In particular, the complexity of EEG signals has been extensively analyzed in the study of neurodegenerative diseases, particularly in AD, showing a reduction in EEG signal complexity in AD patients compared to healthy subjects [33]. However, a systematic study on this topic is still lacking and no method could be considered as a consolidated diagnostic tool.

**Figure 1:**
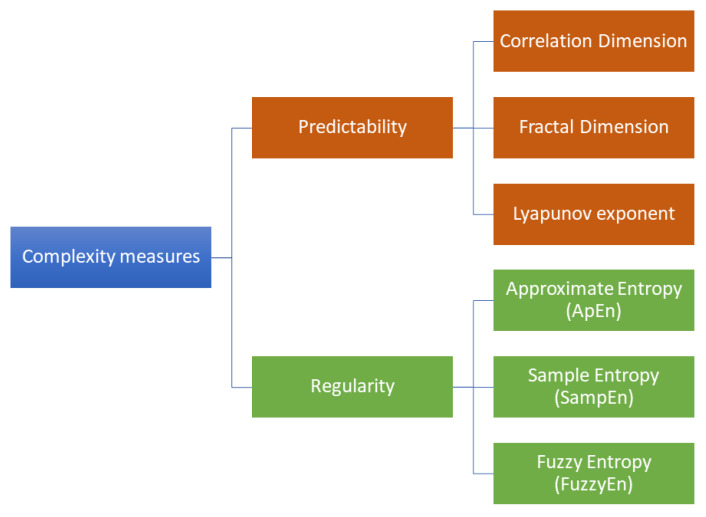
Classification of the most common complexity measures in neurodegenerative disease studies. Predictability measures estimate the temporal and spatial evolution of a dynamic system, whereas regularity measures take into account the presence of repeated patterns along the time series.

The study of complexity has involved evaluating the predictability (through measures of spatial or temporal dimensionality) and regularity (through pattern identification) of EEG signals [33]. Predictability measures describe the temporal and spatial evolution of a dynamic system, while regularity measures take into account the presence of repeated patterns along the time series. Figure 1 provides a general classification of the most common complexity measures. In past studies [42,43], predictability metrics such as Correlation Dimension, fractal dimension, and Lyapunov exponent were used to compare the effects of AD with other forms of dementia and Parkinson’s disease. Despite the observed differences, the precise mechanisms behind the reductions in complexity associated with these conditions are still being debated. It should be noted that predictability measures require a complete reconstruction of the spatiotemporal dimensionality of the signal. On the other hand, regularity measures investigate a lower level of details in the time-frequency domain, in favor of higher robustness. For this reason, they are applicable to noisier or smaller time series (which are typical of biosignals), for which predictability measures could not be reliably calculated [33].

Therefore, in recent years, new metrics for measuring regularity have been proposed. The most commonly used regularity metric is *entropy*, which is a class of statistical measures of the level of uncertainty in a system. In particular, approximate entropy (ApEn), sample entropy (SampEn), and fuzzy entropy (FuzzyEn) are the most investigated in neurodegenerative disease studies. The main differences between these three formulations of entropy are reported in Table 1. ApEn, SampEn, and FuzzyEn arise as single-scale metrics, deducing the degree of complexity from the signal at the original time scale. The basic idea of these entropy measures is to estimate signal complexity by searching for repeated sequences along the entire time series. Traditional implementations operate a binary mechanism of full membership (in-or-out paradigm) [44]. In particular, ApEn and SampEn use the two-state step function (also known as the Heaviside function) to evaluate similarities between patterns by setting a threshold value. Accordingly, two vectors are declared either similar or not. On the other hand, FuzzyEn employs a fuzzy membership function, which returns a real number in the range [0,1] as an output [45]. In this way, two vectors are considered similar according to a continuous degree of similarity. However, biological signals have different meanings at various temporal and spatial dimensions. For this reason, neural complexity can be assessed across different spatial and temporal scales [40]. At the spatial level, neural complexity can be characterized by the complexity of activity patterns within individual neurons or populations of neurons in specific brain regions. At the temporal level, neural complexity can be determined by the complexity of activity patterns over time. Therefore, the EEG exhibits a correlation between multiple spatial and temporal scales.

Since the proposed method is intended solely for the analysis of the EEG signal at each electrode, without considering the various areas of the brain, it may overlook spatial resolution and focus only on evaluating the time scale. For this reason, the single-scale method may not be able to capture the complexity of biological systems [33]. In fact, most physiological signals, such as EEG, reveal different behaviors and patterns over multiple temporal scales. This motivates the introduction of multi-scale entropy measures [46], which can evaluate signal complexity across different time scales. A multi-scale entropy algorithm utilizes one of the traditional entropy formulations (e.g., SampEn) and calculates its value for each resolution scale.

## 3. Complexity Measures through Multi-Scale Entropy

As mentioned in Section 2, neural complexity can be assessed across different spatial and temporal scales. Considering this, Costa et al. [46] proposed a multi-scale entropy measure. Indeed, the multivariate entropy measures, which are statistical techniques used to assess the amount of information or complexity in a system, can be especially useful for studying brain complexity across multiple spatial and temporal scales.

Several studies have exploited multi-scale entropy over time to find a correlation between brain complexity and neurodegenerative diseases, in particular AD, considered the most prevalent neurodegenerative disease. Park et al. [47] conducted a study on the multi-scale sample entropy (MSE) of EEG data from patients with different severities of AD. The study revealed that quantifying the complexity level at various temporal scales provides a dynamic description of AD development. In particular, the EEG data from severe AD patients showed a loss of complexity over a wide range of time scales. These findings were confirmed by Yang et al. [48] and Fan et al. [49], demonstrating that MSE was sensitive to the degree of AD disease, with entropy considerably reducing from moderate to severe AD, as opposed to early-stage AD, when entropy was almost indistinguishable from healthy controls. These complexity decrements could suggest information processing deficits and/or diminished brain dynamics in response to external stimuli. Moreover, these phenomena might involve the loss of functional connectivity, following neuronal death. Mizuno et al. [50] observed decreased entropy at small time scales in frontal areas, consistent with other studies, but also an increased complexity at larger time scales in various brain areas, which could be related to an underlying disconnection phenomenon.

Recently, multi-scale fuzzy entropy (MFE) was introduced; it evolved from the traditional FuzzyEn approach [51]. More specifically, fuzzy entropy is based on the concept of fuzzy logic, which assigns degrees of truth to propositions. The standard set of truth-values ranges from 0 to 1, where 0 represents "totally false," 1 represents "totally true," and values in between refer to partial truth. This multipurpose logic is an extension of Boolean logic, which only uses the values 0 and 1. In the EEG scenario, fuzzy entropy can be applied by considering a template of the signal and identifying other templates that are similar throughout the entire time series. Each time, a value between 0 and 1 will be associated with the degree of similarity or repeatability. This approach can be used to interpret the patterns and corresponding complexity of the brain’s electrical signals. Additionally, MFE takes into account the signals over different time scales and quantifies how "fuzzy" or unpredictable they are at each scale. This provides insights into the organization and coordination of brain activity, which is useful for understanding how different conditions affect brain processes. However, as previously mentioned, FuzzyEn employs a fuzzy membership function, which returns a real number in the range [0,1] as output, considering two similar vectors, according to a continuous degree of similarity. In particular, given a time series x(t) consisting of *N* samples:

(1) x(t)={x(i):1≤i≤N},

a first vector $X_{i}^{m}$ of *m* consecutive samples extracted from x(t):

(2) $$X_{i}^{m}=\left\{x\left(i\right),x\left(i+1\right),\cdots ,x\left(i+m-1\right)\right\}-x_{0}\left(i\right),$$

where *i* is the generic time point where the pattern begins and $x_{0}\left(i\right)$ is the mean value of all *m*-selected samples. Taken $X_{i}^{m}$ and the shifted vector $X_{j}^{m}$ sliding along the signal, the distance $d_{ij}^{m}$ is defined as the maximum absolute difference of the corresponding scalar components of the two vectors:

(3) $$d_{ij}^{m}=max\;\left|\left(x\left(i+k\right)-x_{0}\left(i\right)\right)-\left(x\left(j+k\right)-x_{0}\left(j\right)\right)\right|,$$

where i≠j and k∈{0,m−1}. Then, the similarity degree $D_{ij}^{m}$ of $X_{j}^{m}$ to $X_{i}^{m}$ is calculated as:

(4) $$D_{ij}^{m}=\mu \left(d_{ij}^{m},n,r\right)=\exp\left(\frac{-{\left(d_{ij}^{m}\right)}^{n}}{r}\right),$$

where $\mu (d_{ij}^{m},n,r)$ is the fuzzy membership function.

Typically, $\mu (d_{ij}^{m},n,r)$ is chosen as an exponential function to fulfill two requirements: it has to be continuous without abrupt variations and it has to maximize self-similarity [45]. In Equation (4), *n* and *r* are two arbitrarily assigned parameters, which tune the smoothness of the exponential fuzzy function.

Consequently, the mean over all of the different sequences of length *m* is calculated as follows:

(5) $$\phi^{m}\left(n,r\right)=\frac{1}{N-m}\sum_{i=1}^{N-m}\left(\frac{1}{N-m-1}\sum_{j\neq i,j=1}^{N-m-1}D_{ij}^{m}\right).$$

Similarly, computations are repeated for a second vector $X_{i}^{m+1}$ of length m+1, obtaining the mean $\phi^{m+1}$ from Equation (5). Finally, FuzzyEn can be estimated as the negative natural logarithm of the deviation between $\phi^{m}$ and $\phi^{m+1}$:

(6) $$FuzzyEn\left(m,n,r\right)=\ln\phi^{m}\left(n,r\right)-\ln\phi^{m+1}\left(n,r\right).$$

Hence, FuzzyEn represents the conditional probability that patterns identified for *m* points remain similar for the next (m+1) points.

Defined in these terms, FuzzyEn is a single-scale measure that is not sensible enough to analyze the dynamic processes of the brain. In light of this, it is reasonable to compute FuzzyEn by using a multi-scale approach. In this regard, MFE enables the estimation of the signal complexity over a time-scale interval [52]. In this way, it is possible to search for additional information about the signal by using different time scales.

Starting from the original time series x(t), the basic idea is to change the time scale to recompute the chosen entropy formulation each time:

(7) $$y_{s}\left(t\right)=\sum_{i=j}^{j+s-1}x\left(i\right),\;\mathrm{for}\;1\leq j\leq N-s+1,$$

where $y_{s}\left(t\right)$ represents the new time series at the *s*-th scale factor. In particular, it is desirable to consider at least 15 scale factors [52].

## 4. Possible Practical Implementation, Open Challenges, and Future Perspectives

**Figure 2:**
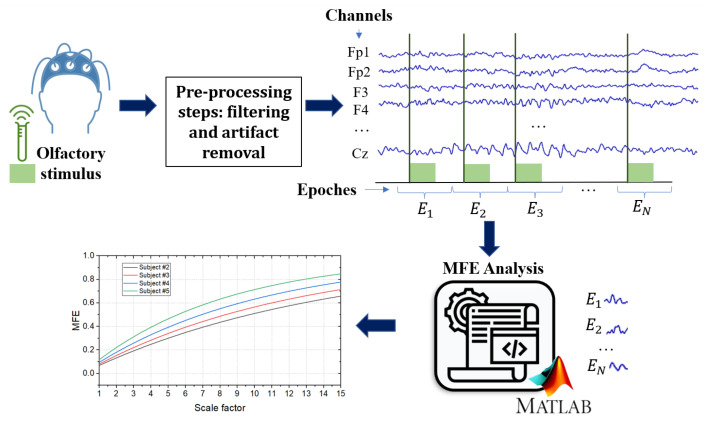
General layout of the proposed approach. The EEG signals are acquired from the subject during an olfactory stimulation task. The acquired signals are filtered with a bandpass filter and an artifact removal technique is performed to remove artifacts. The MFE algorithm was applied to each epoch for each channel.

As mentioned in previous sections, the aim of this work is to discuss and outline the implementation of an entropy-based method for detecting possible common EEG biomarkers between AD and COVID-19 by leveraging olfactory stimulation. Thus, this paper proposes a design of experiments to distinguish EEG data from healthy subjects, COVID-19 patients, and AD patients at different stages of the disease by calculating Multi-scale Fuzzy Entropy (MFE), a scarcely explored formulation of entropy. Figure 2 shows the general layout of the proposed approach.

Considering that olfactory loss occurs early in both AD and COVID-19 as a symptom, the first step is to acquire EEG signals in response to a specific olfactory stimulus. The presence of the stimulus is of fundamental importance in assessing the responsiveness of the brain areas involved in the olfactory task. In this regard, the oddball paradigm is most widely used for an olfactory recognition task. It involves the participant being presented with a stimulus sequence consisting of two different odors, one presented more frequently (non-target) and the other rarely (target) [53]. The choice of fragrances to be used is crucial, as the selected odors should not stimulate the trigeminal system. This is due to the interconnection between the olfactory and trigeminal systems, which can interact and amplify or inhibit each other when exposed to certain stimuli [54,55].

Another critical issue concerns the recording of EEG signals with an appropriate reference, as the electrical potential in the EEG acquisition reflects the difference between two recording sites (electrodes). Hence, it is essential to consider the position and contribution of signals in a reference when analyzing the amplitudes and time differences in quantitative EEG analysis [56]. Specifically, the choice of EEG reference has a significant impact on complexity measures, as discussed in [57], where four different EEG references were compared: linked-mastoids (LM) reference, average (AVG) reference, Laplacian (LAP) “reference-free” transformation, and infinity (INF) reference. The LM reference method calculates the mathematical average of signals from electrodes placed at the mastoids of ears, while the AVG reference method subtracts the average of signals from all electrodes from each individual electrode. The LAP method transforms the raw EEG potentials into a measure of radial current density at the scalp, and the INF reference method approximately transforms a scalp point reference to a reference point at infinity. The findings in [57] suggest that the LAP reference method should be preferred for measuring complexity as it has a positive impact on EEG signal quality and reduces volume conduction effects. In contrast, the LM reference method should be avoided in the context of EEG complexity analysis due to its higher noise levels and tendency to induce artifactual correlations among scalp electrodes. Once the olfactory EEG signal is acquired, it is good practice to pre-process the data to reduce artifacts (e.g., eye blinks) and noise [58]. Typically, a notch filter is used to eliminate power supply interference. Then, several techniques may be employed to minimize artifacts, depending on the number of channels used to acquire the EEG signal [59]. The resulting signal can be segmented into epochs centered on the olfactory stimulus, considering seconds of pre- and post-stimulus. Finally, the data could be used for an MFE analysis by considering a time-scale range (e.g., 15 scale factors [52]). Moreover, it could be interesting to investigate the complexities of signals at different frequency bands. In this instance, the frequency bands delta (0.5–3 Hz), theta (3–8 Hz), alpha (8–13 Hz), beta (13–30 Hz), and gamma (30–45 Hz) might be used to divide EEG signals and then apply the MFE algorithm to each frequency range.

However, there are several open challenges and future perspectives in the study of the complexity of EEG olfactory signals to discriminate or identify common features among different diseases (such as COVID-19 and AD).

An open issue is the lack of clinical standards for entropy measures on EEG signals, making entropy-based results across different studies difficult to compare [33]. As previously mentioned, entropy measures such as SampEn and ApEn have been used for many years in research to assess the complexity of brain signals. However, there is a limited understanding of the relationship between different entropy measures and the underlying brain processes they are intended to represent. Moreover, different algorithms exhibit significant variability, which can lead to inconsistent results and incorrect interpretations. The selection of inherent parameters for each entropy formulation is a crucial source of variability. Parameters such as the sliding window length on the time series or the threshold set to define similarity between signal patterns can significantly influence the results of an entropy analysis. Although some values are commonly used in several studies, the parameters must be adjusted for each individual application. To overcome this problem, a systematic study with a sufficient number of subjects at various stages of disease progression would be necessary.

Another major issue related to the lack of clinical standards involves the lack of available data. Despite the extensive use of EEG for mapping brain activity both in clinical and research contexts, the limited availability of public datasets is still a major drawback. Although over the years, this lack of open-access data has been addressed [60,61], it is still challenging to find suitable data online. Given the significant amount of EEG recordings collected worldwide every year, it is fair to assume that only a small portion of the potentially existing data is made available.

A main aspect to consider is certainly the concern regarding the sensitive information contained in EEG recordings. When sharing this type of sensitive data online, especially data regarding the health conditions of individuals, there are specific ethical and privacy regulations that must be respected. Therefore, most research groups are discouraged from sharing such data from their experiments.

Moreover, collecting and preparing an EEG dataset for public use can be challenging for several reasons. It often requires expensive instrumentation to perform time-consuming experiments, which must be well-planned and conducted in a suitable environment. Additionally, due to the lack of generally recognized standards, experiments depend on many design choices. The process of EEG data recording strongly depends on the working conditions of the specific application or research focus. Hence, first-hand collected data can be biased, limiting the reproducibility of the experiment and compromising the generality of the results.

Few large datasets have been offered by past BCI competitions [62]. However, they mainly contain EEG data from healthy subjects engaged in specific activities, such as motor-imagery tasks. Other EEG datasets can be found on open-access platforms, such as OpenNeuro [63] and PhysioNet [64]. Nevertheless, comprehensive open-access EEG datasets with both healthy subjects and patients with neurodegenerative diseases are still lacking in EEG-based research.

As mentioned in the previous sections, neuronal complexity can be analyzed on both multiple spatial and temporal scales. This work focuses on the use of MFE, which analyzes the EEG signal at multiple time scales. However, since the proposed method is intended only for the analysis of the EEG signal at each electrode, without considering the various areas of the brain, it is possible to overlook the spatial resolution and focus only on the evaluation of the time scale. Metrics that analyze the signal in spatial dimensions or in spatial and time dimensions simultaneously can also be taken into account. An example of this is neural complexity measures that directly quantify neural interactions across local and global scales. The key idea of neural complexity is the coexistence of functional specialization and integration [40]. The former refers to the idea that different regions of the brain are specialized in processing specific types of information or performing specific functions. Functional integration, on the other hand, refers to the coordination and communication between different specialized regions of the brain to achieve more complex functions. This involves integrating information from different sources and synchronizing activity across multiple brain regions. Understanding functional specialization and integration is crucial for comprehending brain function and behavior. Impairment of either of these aspects can result in neurological and psychiatric disorders. Therefore, it may be worthwhile to evaluate neural complexity across spatial and temporal scales to gain insights into the underlying mechanisms of neural processing.

Finally, machine learning (ML) approaches are being increasingly used to identify features extracted from EEG signals acquired during olfactory tasks and to diagnose diseases. In the literature, ML methods have been implemented to investigate the physiology of pattern-based odor detection and recognition processes, as well as develop classifiers for diseases based on biomarkers that include olfactory features [65]. However, despite these efforts, it is still difficult to create a classifier for the diagnosis of olfactory dysfunctions, often related to silent diseases such as neurodegenerative diseases. Therefore, greater efforts in the field of ML need to be conducted. Currently, signal processing is mostly based on the extraction of features in the time, frequency, and spatial domains, such as power spectral density or complexity-based features [66,67]. Nevertheless, the most commonly used pattern recognition approaches are based on principal component analysis (PCA) or linear discriminant analysis (LDA) [67]. Hence, there is a necessity to enhance ML algorithms that could aid in the development of a system to identify olfactory deficits and specific diseases.

In light of this, it would be highly beneficial to explore the potential of ML algorithms in conjunction with the complex analysis of EEG signals to differentiate various diseases. In particular, developing an ML framework based on entropy features extracted from EEG signals could facilitate the classification of diseases, including AD and COVID-19. The fundamental concept is to extract relevant characteristics, such as entropy features, from the EEG signals associated with a specific disease. In this way, similar to its successful applications in other medical domains, such as [68,69,70], the utilization of artificial intelligence, including machine and deep learning, may enhance diagnostic accuracy.

## 5. Conclusions

In this paper, we highlighted emerging studies on the correlation between COVID-19 and neurodegenerative diseases. In more detail, we showed that neurological mechanisms of proteins interaction during COVID-19 are similar to those exhibited in AD. Hence, it is necessary to quantify the commonalities between AD and COVID-19 and how similar the effects they produce on the human nervous system are. Moreover, as shown in the literature, olfactory system impairment may occur in both SARS-CoV-2 infection and AD. In this regard, measuring olfactory performance through EEG signals could be used to explore common biomarkers between these two diseases. In particular, analyzing the complexity and irregularity of neuronal activities of the brain is extremely important in identifying the process of cognitive impairment. To this end, this paper presents an overview of the use of multi-scale entropy analysis to investigate brain complexity and provide an objective evaluation of these issues. Entropy analysis is expected to have a more significant future impact when combined with traditional techniques, such as event-related potentials and time-frequency analysis, especially as a support in clinical diagnostics.

Beginning with these considerations, it would be interesting to investigate the possible common EEG entropic features between COVID-19 and neurodegenerative diseases (such as AD) when subjected to an olfactory task. For this reason, this perspective paper proposes a practical implementation of an MFE-based method by leveraging olfactory stimulation. In this context, open challenges and future perspectives are outlined. In light of this, future work will be dedicated to testing the proposed procedure on a large-scale campaign on voluntary subjects, divided into the following categories: healthy subjects, AD patients, and people with a history of COVID-19. The proposed procedure could be applied to the EEG patterns of each patient, calculating the MFE values for each channel and each EEG band. In this way, it could be determined if the AD and COVID-19 cases exhibit a similar trend, with different values with respect to the healthy subjects. Such a result could highlight that similar neurodegenerative phenomena may be implicated in the two diseases, opening unexplored avenues in which further research will be needed.

## Figures and Tables

**Table 1:** Advantages and limitations of the three formulations of single-scale entropy.

Entropy	Similarity Criterion	Advantages	Limitations
ApEn	Two-state step function (Heaviside function) with a fixed threshold.	It exploits a template-wise approach to identify patterns and regularities. It can be applied to noisy short signals.	It is heavily dependent on recorded data and chosen values for parameters. It counts as self-matches.
SampEn	Two-state step function (Heaviside function) with a fixed threshold.	It is robust to noise and non-stationarity. It does not count self-matches.	It is heavily influenced by chosen values for parameters.
FuzzyEn	Continuous degree of similarity based on an exponential membership function.	It estimates the degree of uncertainty (fuzziness) of a signal. It is highly insensitive to noise but it is sensitive to complexity. It does not count self-matches.	It can be affected by small changes in the degree of membership. It requires more computational steps.

## Data Availability

No data were reported in this study.

## Abbreviations

The following abbreviations are used in this manuscript:

AD	Alzheimer’s disease
ApEn	approximate entropy
BBB	blood–brain barrier
CNS	central nervous system
EEG	electroencephalography
FuzzyEn	fuzzy entropy
MCI	mild cognitive impairment
MFE	multi-scale fuzzy entropy
ML	machine learning
MSE	multi-scale sample entropy
PCA	principal component analysis
SampEn	sample entropy
SARS-CoV-2	severe acute respiratory syndrome coronavirus 2
SSE	Shannon’s spectral entropy
